# Mosaic TLR8 gain-of-function in an elderly female treated with JAK inhibitor and adjunctive therapy

**DOI:** 10.70962/jhi.20260113

**Published:** 2026-09-24

**Authors:** Jenny A. Patel, Nermina Saucier, Hwi M. Gil, Elizabeth G. Schmitz, Marianna B. Ruzinova, Ayah Saffaf, Morey Blinder, Neha Mehta-Shah, Zachary D. Crees, Megan A. Cooper, Ofer Zimmerman

**Affiliations:** 1 Division of Rheumatology and Immunology, Department of Pediatrics, Washington University in St. Louis, St. Louis, MO, USA; 2 Division of Allergy and Pulmonary, Department of Pediatrics, Washington University in St. Louis, St. Louis, MO, USA; 3 Department of Pathology and Immunology, Washington University in St. Louis, St. Louis, MO, USA; 4 Division of Allergy and Immunology, Department of Medicine, Washington University in St. Louis, St. Louis, MO, USA; 5 Division of Hematology, Washington University in St. Louis, St. Louis, MO, USA; 6 Division of Oncology, Washington University in St. Louis, St. Louis, MO, USA

## Abstract

We report severe neutropenia in a 77-year-old female with mosaic TLR8 gain-of-function due to a novel variant. Treatment with ruxolitinib, prednisone, and GM-CSF resulted in clinical improvement. This case expands the phenotypic spectrum beyond pediatric and male cohorts, highlighting mosaicism in late-onset immune dysregulation.

## Teaser

Patel et al. describe an elderly woman with severe chronic neutropenia caused by mosaic TLR8 gain-of-function. The report broadens a primarily pediatric, male disease spectrum and highlights somatic mosaicism as an important diagnostic consideration in adults with unexplained cytopenia and immune dysregulation.

## Introduction

The majority of genetic errors of immunity (GEI) are driven by inherited germline defects. Somatic mosaicism—pathogenic genetic changes arising post-zygotically in a subset of cells—is increasingly recognized as a clinically relevant and underappreciated mechanism of monogenic GEI (1). We recently described somatic and germline TLR8 gain-of-function (GOF) as an X-linked dominant GEI characterized by severe neutropenia, lymphoproliferation, hypogammaglobulinemia, and bone marrow failure (2).

TLR8 is an X-linked endosomal pattern recognition receptor expressed on myeloid cells that senses viral and bacterial single-stranded RNA, signaling through NF-κB and related pro-inflammatory pathways (2, 3). Originally described in males, five of six boys had somatic mosaicism with peripheral blood variant allele frequencies (VAF) < 30% (2). Our recently expanded cohort of 10 patients defined a broader clinical spectrum, with an age of symptom onset ranging from 9 mo to 28 years, including the first female patient who presented in infancy with germline disease (3). There have been no reports of adult or mosaic female patients. Here, we describe an elderly female with chronic severe neutropenia and invasive fungal pneumonia with a novel GOF *TLR8* mosaic variant.

## Clinical presentation

**Figure 1.**
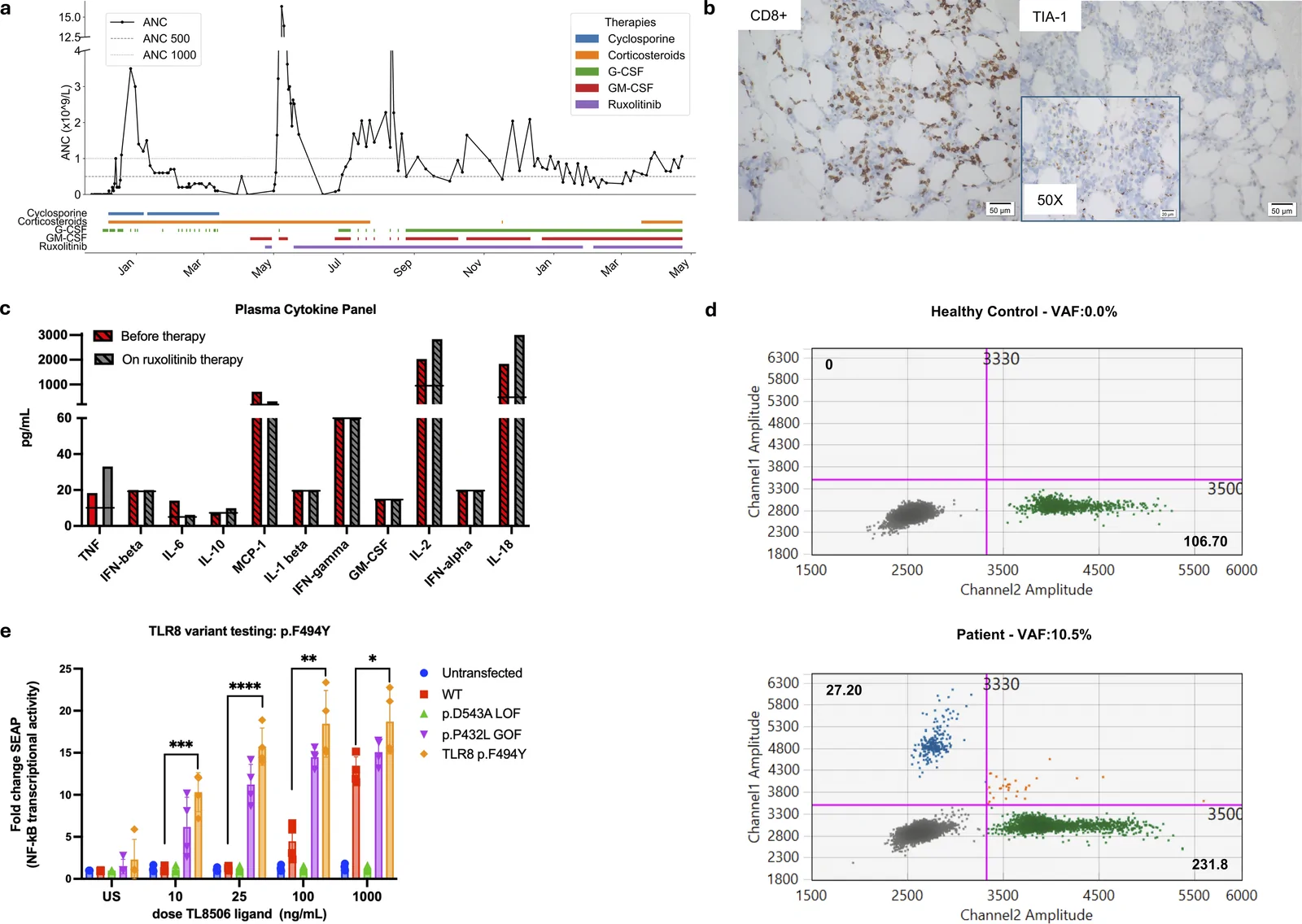
**Longitudinal treatment response, marrow findings, cytokine profile, and functional confirmation of *TLR8* p.Phe494Tyr as a gain-of-function variant. (a)** Patient ANC and different therapies over time. Dashed line represents level of 500 cells per µl, and dotted line represents level of 1,000 cells per µl. **(b)** Patient bone marrow staining for CD8^+^ cells and T cell intracellular antigen-1 (TIA-1) expression, highlighting patient’s high level of marrow cytotoxic T cells. **(c)** Patient serum cytokine levels (Mayo Clinic, MN, USA) before and during immunomodulator therapy. Black horizontal lines represent the highest normal level of each measured cytokine. **(d)** Droplet digital PCR (ddPCR) analysis of the *TLR8* p.F494Y variant allele frequency (VAF) in genomic DNA extracted from PBMCs of a healthy control (top panel) and the patient (bottom panel), PBMCs extracted genomic DNA. **(e)** NF-κB reporter cells (HEK-Blue Null1 cells) that do not express endogenous TLR8 were transfected with WT *TLR8*, patient *TLR8* variant (encoding p.F484Y), a known GOF variant (encoding p.P432L), or a loss-of-function (LOF) *TLR8* variant (encoding p.D543A) and stimulated with the indicated doses of the TLR8-specific agonist TL8-506 for 24 h, as published before (2). Patient *TLR8* variant leads to GOF in TLR8 activity as measured by NF-κB transcriptional activity. Data are represented as mean ± standard deviation of biological replicates and representative of four independent experiments (TL8-506). *P < 0.05, **P ≤0.01, ***P ≤0.001, and ****P < 0.0001 by two-way ANOVA test.

A 77-year-old female with a history of right breast ductal carcinoma treated with lumpectomy, obstructive sleep apnea, and a 20-year history of neutropenia was referred for immune evaluation following hospital admission for pneumonia. The patient was enrolled in an Institutional Review Board–approved study after written informed consent at Washington University School of Medicine. Prior to this infection, despite persistently low absolute neutrophil counts (ANC) between 0.0–0.3 K/cumm (reference [ref.] range: 1.50–6.50 K/cumm) (Fig. 1 a), she had no life-threatening infections. She did report episodes of otitis media, skin and soft tissue infections with cellulitis, a perianal abscess with fistula, and a dental abscess, all responding to antimicrobials.

Five bone marrow biopsies performed over a period of 10 years showed normocellular or mildly hypercellular marrow for age without dysplasia or evidence of myelodysplastic syndrome or leukemia. Karyotype was 46, XX. Each biopsy did demonstrate small infiltrates of CD8-predominant T cells (up to 8% of cellularity) (Fig. 1 b). Three targeted next-generation sequencing (NGS) myeloid malignancy panels over the course of 3 years revealed two *TET2* frameshift/stop gain variants with VAFs between 0.8 and 6% and a *STAT3* missense pathogenic variant (p.S614R) with a VAF of 0.15–1%. The *TET2* variants were consistent with clonal hematopoiesis frequently observed with aging. Blood and marrow TCR-γ sequencing demonstrated polyclonal rearrangements without malignancy. A population of morphologically large granular lymphocytes was observed in a peripheral smear, with consideration of large granular lymphocytic leukemia; however, the lack of a clonal T cell receptor rearrangement did not support this diagnosis. Antineutrophil antibodies were negative. Targeted genetic sequencing (Invitae inborn errors of immunity and cytopenias panel, CA, USA) identified two *UNC13D* variants in cis: one pathogenic (c.177_178del [p.Tyr61Hisfs*10]) and one of uncertain significance (c.197G>A [p.Arg66His]), suggesting carrier status for autosomal recessive hemophagocytic lymphohistiocytosis (HLH), supported by normal natural killer cell degranulation and lack of clinical features of HLH. Additional immune studies showed lymphopenia with low absolute level of CD4 (133, ref. 365–1,294) and CD8 (161, ref. 187–781) T cells, natural killer (35, ref. 76–467) cells, undetectable B cells, and an elevated level of CD4^−^CD8^−^ double negative T cells (10%; ref.: <5%). Initially, she had normal total immunoglobulin levels, and protective anti-tetanus and anti-*Streptococcus pneumoniae* IgG antibodies. Serum cytokine panel (Mayo Clinic, MN, USA) demonstrated elevated levels of TNF, IL-6, MCP-1, IL-2, and IL-18 (Fig. 1 c).

For over 20 years, she was intermittently treated with methotrexate, cyclosporine, intravenous immunoglobulin, granulocyte colony-stimulating factor (G-CSF), and/or GM-CSF, without significant improvement of her ANC.

At age 77, she developed invasive *Cladosporium* fungal pneumonia. Her ANC was 0.0 K/cumm, and due to inability to effectively mount adequate immune response for clearance of the invasive fungal infection without neutrophils, she received prednisone, cyclosporine, and GM-CSF. She had a transient rise in ANC to 3.5 K/cumm (Fig. 1 a), and the infection cleared on antifungals.

A targeted clinical somatic mosaicism panel for inborn errors of immunity (nine genes: *FAS*, *JAK1*, *KRAS*, *NRAS*, *PIK3CD*, *PIK3R1*, *STAT5B*, *TLR8*, and *UBA1*; mean sequencing depth >=4,000×; Washington University in St. Louis, St. Louis, MO, USA) was performed on whole blood genomic DNA and identified a mosaic missense variant in *TLR8*, (chr17:42322402:C>A, c.1981G>T, p.Phe494Tyr), with a VAF of 12.1%, confirmed by research-based Droplet digital PCR (ddPCR) (Fig. 1 d). This novel variant (ClinVar ID 4279980) was not present in the gnomAD database. In vitro studies showed enhanced TLR8 signaling via elevated NF-κB in HEK-Blue Null1 cells (InvivoGen) transfected with the *TLR8* p.Phe494Tyr variant, stimulated with TL8-506 (Fig. 1 e, methods [2]), establishing GOF pathogenicity as the etiology of her bone marrow failure with severe neutropenia. X chromosome inactivation analysis (Greenwood, SC, USA) demonstrated an X-inactivation ratio of 95:5, consistent with a highly skewed X-inactivation pattern, although it is uncertain if this is due to the *TLR8* variant or a consequence of aging due to a reduced hematopoietic pool, as evidenced by clonal hematopoiesis found in this woman in her 70s.

A trial of ruxolitinib was initiated to suppress inflammatory cytokine signaling in myeloid and lymphoid cells, which may lead to decreased activation and exhaustion of hematopoietic progenitors in TLR8-driven disease. She remained on GM-CSF and G-CSF growth factors, intermittent courses of prednisone, and prophylactic antimicrobials. This combination of ruxolitinib 5 mg BID, prednisone 5 mg daily, and weekly GM-CSF and G-CSF was effective in maintaining patient’s ANC above 500 cells/µl (Fig. 1 a). At the time of genetic diagnosis, she developed hypogammaglobulinemia (IgG 508 mg/dl [ref. 700–1,600]) and after hospitalization for cellulitis was started on immunoglobulin replacement therapy. Hematopoietic stem cell transplantation (HSCT) has been considered, but she carried substantial risks due to her age and clinical status, and through shared decision-making, HSCT was deferred.

## Discussion

This case represents the oldest patient reported with TLR8 GOF and the first female with likely somatic mosaicism. The possibility of gonosomal mosaicism cannot be ruled out by testing blood. However, based on her age at presentation the mechanism is most consistent with somatic mosaicism of the hematopoietic compartment. It raises several important clinical and biologic points that extend the existing literature.

*TLR8* is encoded on the X chromosome, and in males, mosaicism produces a hemizygous population of cells carrying the GOF allele alongside cells carrying a WT population. Even when fewer than 30% of the cells carry the variant, significant disease develops (3). Due to the X-linked dominant mechanism in this disease, a heterozygous mosaic state in a female is also disease causing. *TLR8* generally escapes X-inactivation, but this may vary between individuals, cell types, and with age (4). Our patient demonstrated highly skewed X chromosome inactivation, which may have contributed to the worsening of her TLR8 GOF phenotype over time.

The mechanism(s) by which reported *TLR8* variants lead to GOF are uncertain. However, a recent study from the Gantier laboratory identified a TLR8 antagonist binding site that recognizes short RNA degradation fragments of 1–3 bases, including endogenous RNA, to stabilize an inactive conformation and prevent receptor activation (5). The amino acid affected in the patient here, F494, is located at this antagonist binding site. They demonstrated that another missense variant at this same amino acid reported in our initial cohort (3) (p.F494L) was resistant to antagonism (5). Therefore, the F494Y variant may impair the natural antagonism that dampens WT TLR8 activity, potentially conferring TLR8 resistance to inhibition.

The diagnostic challenges in TLR8 GOF are severalfold. First, standard clinical NGS panels are designed for germline variant detection and often filter out low-level mosaic variants (2, 3). Second, due to TLR GOF disease’s ability to mimic other well-established conditions (e.g., B cell lymphopenia with antibody deficiency, high number of double-negative T cells as often seen in autoimmune lymphoproliferative syndrome), a patient can be misdiagnosed.

Similar to previously described TLR8 GOF patients, this patient’s neutropenia was unresponsive to colony-stimulating growth factors and immunosuppressive therapies. However, the combination of ruxolitinib with prednisone, GM-CSF, and G-CSF was effective in keeping patient ANC above 0.5 K/cumm, with no hospitalizations over 12 mo follow-up. Interestingly, ruxolitinib had no significant effect on patient's elevated cytokines (Fig. 1 c), despite the clear improvement in her neutrophil count under this therapy (Fig. 1 a). Available published clinical data suggest that HSCT is currently the only curative option for patients with TLR8 GOF (2, 3). While HSCT may be a feasible option in pediatric patients, this option carries very high risk in patients over 70 years of age. Further investigation is needed to establish optimal management strategies across age groups.

As deep sequencing to detect mosaic variants becomes more widely available clinically and incorporated into the evaluation of refractory cytopenia and immune dysregulation in adults, it is likely that the demographic profile and spectrum of disease with TLR8 GOF will continue to broaden.

### Conclusion

This case underscores that TLR8 GOF is an X-linked dominant disease, which can affect both female and male children and adults. Accurate diagnosis is essential, given the progressive and fatal course of untreated disease and curative potential of HSCT. Immune modulators can be used to manage patients awaiting HSCT or patients in which HSCT is not a viable option.

## Data Availability

The data underlying this study are included in the published article. The *TLR8* p.Phe494Tyr variant was deposited in ClinVar (Variation ID 4279980). Additional patient-level clinical data are not publicly available because of patient privacy considerations but are available from the corresponding author upon reasonable request, subject to applicable institutional and ethical requirements.

## References

[1] Schmitz, E.G., M. Griffith, O.L. Griffith, and M.A. Cooper. 2025. Identifying genetic errors of immunity due to mosaicism. J. Exp. Med. 222:e20241045. 10.1084/jem.20241045 PMC11998702 40232243

[2] Aluri, J., A. Bach, S. Kaviany, L. Chiquetto Paracatu, M. Kitcharoensakkul, M.A. Walkiewicz, C.D. Putnam, M. Shinawi, N. Saucier, E.M. Rizzi, . 2021. Immunodeficiency and bone marrow failure with mosaic and germline TLR8 gain of function. Blood. 137:2450–2462. 10.1182/blood.2020009620 PMC8109013 33512449

[3] Arnold, D.E., S. Kaviany, J. Aluri, K.R. Calvo, S.S. Mehta, J.M. Tobin, L.G. Schuettpelz, N. Saucier, E.G. Schmitz, L. Murguía-Favela, . 2026. Clinical characteristics, management, and hematopoietic cell transplantation of patients with TLR8 gain-of-function. Blood Adv. 10:1967–1976. 10.1182/bloodadvances.2025016338 PMC12997320 41370196

[4] Youness, A., C. Cenac, B. Faz-López, S. Grunenwald, F.J. Barrat, J. Chaumeil, J.E. Mejía, and J.-C. Guéry. 2023. TLR8 escapes X chromosome inactivation in human monocytes and CD4^+^ T cells. Biol. Sex Differ. 14:60. 10.1186/s13293-023-00544-5 PMC10506212 37723501

[5] Alharbi, A.S., S. Sapkota, Z. Zhang, R. Jin, E. Rupasinghe, W.S.N. Jayasekara, D. Yu, M. Speir, L. Wilkinson-White, L. Cubeddu, . 2026. 2'-O-Methyl-guanosine RNA fragments antagonize TLR7 and TLR8 to limit autoimmunity. Nat. Immunol. 27:762–775. 10.1038/s41590-026-02429-2 PMC13043311 41667621

